# The effectiveness of physiotherapy for patients with isolated cervical dystonia: an updated systematic review and meta-analysis

**DOI:** 10.1186/s12883-023-03473-3

**Published:** 2024-02-01

**Authors:** Shimelis Girma Kassaye, Willem De Hertogh, David Crosiers, Esayas Kebede Gudina, Joke De Pauw

**Affiliations:** 1 Institute of Health, Jimma University, Jimma, Ethiopia; 2 Department of Rehabilitation Sciences and Physiotherapy/Movant, Faculty of Medicine and Health Science, University of Antwerp, Antwerp, Belgium; 3 Department of Neurology, Antwerp University Hospital, Antwerp, Belgium; 4 Translational Neurosciences, Faculty of Medicine and Health Sciences, University of Antwerp, Antwerp, Belgium

**Keywords:** Cervical dystonia, Torticollis, Physiotherapy, Physical therapy

## Abstract

**Background:**

Cervical dystonia is a movement disorder typically characterized by a patterned and twisting movement of sustained or intermittent muscle contractions. Recently, new clinical trials are emerging, highlighting the potential benefit of physiotherapy (PT) on disease outcomes. Thus, the objective of this review is to update the effectiveness of PT on cervical dystonia disease outcomes and subsequently perform a meta-analysis.

**Methods:**

Interventional studies published in English with adult patients with isolated cervical dystonia following a physiotherapy program were included. Relevant articles were searched in PubMed (MEDLINE), Web of Science, and Scopus. Cochrane and Joanna Briggs Institute risk of bias checklists were used for quality reporting. Meta-analysis was done using Review Manager 5.3 statistical software and a pooled mean difference for pain was presented.

**Results:**

Fourteen articles were included in the review and two articles were included in the meta-analysis. The meta-analysis revealed that PT intervention had a significant effect on pain reduction scale (-5.00, 95% CI -6.26, -3.74) when used as an additional therapy with botulinum toxin (BoNT) injection. Additionally, findings indicate a possible positive effect of PT disease severity, disability, and quality of life.

**Conclusions:**

Physiotherapy in addition to BoNT is recommended to decrease pain. The findings suggest a reduction of disease severity, disability, and improvement in quality of life. The variety in the type and duration of PT interventions did not allow a clear recommendation of a specific type of PT.

## Introduction

Cervical dystonia (CD) is a movement disorder characterized by sustained or intermittent muscle contractions causing abnormal, often repetitive movements, postures, or both. It is typically patterned, twisting, and may be tremulous [1]. Isolated cervical dystonia is the most common form of dystonia with a prevalence estimate of 20 − 4,100 cases/million [2, 3]. Although CD does not affect patients’ life expectancy, the disease is disabling and markedly affects patients’ quality of life [4] by causing severe functional and psychosocial impairment [5].

Functional limitations in different domains have been reported in most patients with CD [6] with impact on mobility-related activities (such as walking, driving, crossing the street, and parking) [7, 8], reduced productivity and job loss [9]. Quality of life (QoL) is also significantly affected by the presence of physical and mental health problems including mood disorders, anxiety, depression, low self-esteem, low self-confidence and pain [10].

The first treatment of choice are intramuscular injections of botulinum toxin (BoNT) [11], repeated every three to four months [12]. Other neurosurgical [13–15] and pharmacological [16] interventions are available but have various drawbacks. Additional to BoNT injection, paramedical interventions such as physiotherapy (PT) are advised. In 2014, we published a systematic review on the effect of PT [17]. Since then, multiple studies have been published, warranting a new overview of the effectiveness.

Therefore, the aim of this review is first to update the knowledge on the effect of PT on patients’ functioning, pain, disease severity, and quality of life. Secondly, to evaluate the effect of PT as add-on to BoNT by conducting a meta-analysis.

## Methods

The Preferred Reporting Items for Systematic reviews and Meta-Analyses (PRISMA 2020) was followed [18]. The protocol was registered in PROSPERO with the number CRD42022376433 on 07/12/2022.

### Eligibility criteria

Included studies are those reporting the effect of a physical therapy program (I) in patients with isolated cervical dystonia (P) on patients’ functioning including pain, disease severity, disability, and quality of life (O). In the review published in 2014 by De Pauw J et al. [17], case reports were included. For this updated literature review, the aim was to include only interventional studies. Therefore, observational studies, case reports, and conference papers were excluded.

For the meta-analysis, studies comparing PT and BoNT with BoNT alone were considered when reporting disease severity by standardized measures such as the visual analogue scale (VAS) for pain, Toronto Western Spasmodic Torticollis Rating Scale (TWSTRS), and standardized QoL outcome measures.

### Information sources

Three databases, PubMed (MEDLINE), Web of Science, and Scopus were searched for potentially relevant studies. The source was last searched on August 22, 2023.

### Search strategy

The search strategy was developed following the PICO framework: population (P): adult patients with isolated cervical dystonia, intervention (I): physical therapy alone or adjuvant to BoNT injections, comparator (C): was not specified to include all available studies and the outcome(s) of interest (O) was: pain, disease severity, disability, or quality of life but was not specified to include all available studies. Accordingly, the following keywords are used: cervical dystonia or spasmodic torticollis combined with keywords related to physical therapy such as rehabilitation, physiotherapy, relaxation therapy, neuromotor rehabilitation, exercise therapy. The search strategy for each database is presented in (Table 1).

**Table 1:** Databases search strategy

Database	Search Strategy
Pubmed	((((“Cervical Dystonia, Primary” [Supplementary Concept] OR “cervical dystonia” [All Fields] OR “spasmodic torticollis” [All Fields])))) AND (((“physical therapy modalities“[MeSH Terms] OR “physical therapy modalities“[All Fields] OR “physical therapy“[All Fields] OR “physiotherapy“[All Fields] OR “neuromotor rehabilitation” [All Fields] OR “Rehabilitation”[MeSH Terms] OR “relaxation therapy” [All Fields])))
Web of Science	TS=(cervical dystonia OR spasmodic torticollis) AND (TS=(physical therapy OR physical therapy modalities OR relaxation therapy OR neuromotor rehabilitation OR rehabilitation OR exercise therapy) NOT TS=(deep brain stimulation)) AND document types: (Article)
Scopus	(“cervical dystonia” OR “spasmodic torticollis”) AND (“physical therapy” OR “physical therapy procedure” OR “neuromotor training” OR “relaxation therapy” OR “neuromotor rehabilitation” OR “rehabilitation”)

### Selection process

Following removal of duplicates, all titles and abstracts of identified articles were independently screened for eligibility by two authors (JDP and SG) on the Rayyan platform [19]. The full texts of potentially relevant studies were then independently assessed to determine eligibility based on the predetermined inclusion and exclusion criteria. In case of discrepancies, a consensus meeting was held.

### Data collection process

Prior to the data collection, a data extraction instrument was designed according to the Cochrane handbook for systematic reviews of interventions [20]. Key data concerning study design, study participants (sample size, sex, mean age), information regarding the disease and medical treatment if provided (duration of cervical dystonia, severity of the symptoms, duration of treatment, medication or BoNT injections), information regarding the intervention (physical therapy modalities, duration and frequency of sessions), and intervention outcomes (pain, disease severity, disability and QoL) from each of the selected studies were extracted independently by two reviewers (JDP and SG). A consensus meeting was held in case of discrepancies.

### Study risk of bias assessment

Risk of bias was independently assessed by two reviewers (JDP and SG) using the Cochrane Risk of Bias checklist for randomized controlled trials [21] and the Joanna Briggs Institute (JBI) checklist for non-randomized experimental studies and case series [22]. A consensus meeting was held in case of disagreement.

### Effect measures and synthesis methods

For the meta-analysis, statistical software Review Manager 5.3 was used. We aimed to estimate the change in the mean score of cervical dystonia symptoms (TWSTRS total score, pain, severity, and disability score) following a physical therapy intervention consisting of multiple treatment modalities. Studies reporting only one treatment option such as tape were not included. The pooled mean difference was presented for the outcome. The Cochrane Q-test and I^2^ statistics were used to evaluate heterogeneity between included studies. The I^2^ value is interpreted as follows: 0–40% (not important); 30–60% (moderate heterogeneity); 50–90% (substantial heterogeneity); and 75–100% (considerable heterogeneity) [23].

## Results

### Study selection

**Fig. 1:**
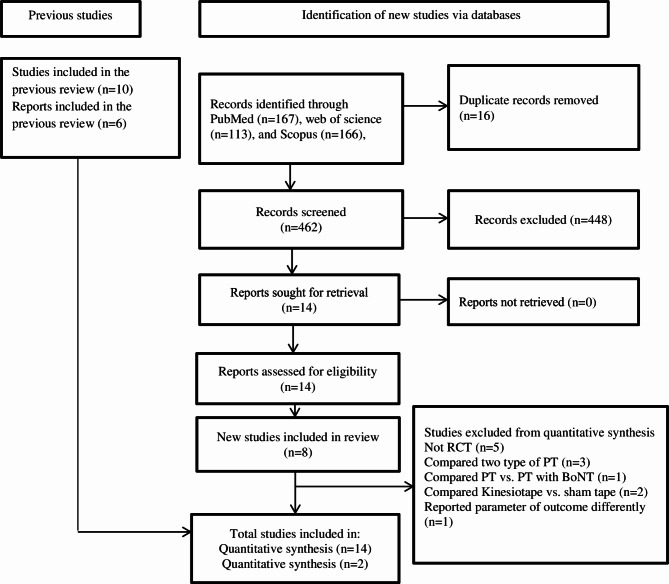
PRISMA flow chart - updated systematic literature review

A total of 466 articles were initially identified. Before screening, duplicates were removed, resulting in 462 reports. In the first screening phase of title and abstract, 488 were excluded. The full texts of 14 potentially eligible reports were retrieved and included as they all met the inclusion criteria. Since the previous review in 2014 by De Pauw et al. [17], 8 new studies have been published and included in this review. Contrary to the review of 2014, case-reports were now excluded. Two of the 14 articles [24, 25] reported the effect of PT intervention on pain and were selected for meta-analysis. The detailed selection process is depicted in the PRISMA flow chart (Fig. 1).

### Study characteristics

Of the 14 included studies, 8 were RCTs [24, 26–32], 3 case-control studies [25, 33, 34], and 3 case-series [35–37]. A total of 414 patients with CD participated in the included studies. The mean reported duration of cervical dystonia ranged from 21.2 months to 27.8 years. Study participants in the intervention group received various physical therapy interventions modalities such as stretching, exercise therapy (posture, coordination, proprioception, strengthening underactive muscles, motor learning, relaxation, sensorimotor exercises with feedback), aquatic relaxation therapy, home exercise program, active and passive mobilizations of the neck, taping, biofeedback and Transcutaneous Electrical Neural Stimulation (TENS). Twelve out of 14 studies [24–28, 30, 31, 33–37] applied various physical therapy modality interventions whereas 2 studies [29, 32] used taping as the sole intervention.

The duration of the physical therapy intervention ranged from two weeks to 12 months. In 11 of the 14 included articles, the physical therapy intervention was additional to BoNT injections. In two studies, none of the participants received BoNT [33, 35]. In one study, about one-third of participants received BoNT during the length of the study [26]. Two studies compared a specialized physiotherapy program to standard physiotherapy care additional to BoNT [27, 31] (Table 2).

**Table 2:** Summary finding of included studies

Author, year of publication	Study design	Patients (M,F)	Duration of CD (year, Mean ± SD)	Intervention (and its duration) for experimental group	Control	Outcome Measures	Follow-up	Result	Include in meta-analysis of PT versus no-PT on disease outcomes
Werner et al., 2019	Monocentric prospective interventional	18 (M = 3, F = 15)	7.5 (± 6.6)	BoNT + PT: 12 sessions of 45 min at ratio of 2x/week (30 min PT session and 15 min TENS application) for a total of 6 months	BoNT alone	Cervical ROM (ZEBRIS), QoL (SF-36), TWSTRS (total, severity, disability, and pain scale)	After 6 weeks (T1) and on the last day of the first phase (T2).	Significant improvement in TWSTRS severity, disability and pain measures after intervention	Yes
Van den Dool, 2019	RCT	72 (M = 30, F = 42)	12.7 (± 10.4)	BoNT + Specialized PT consisting of 30 min sessions of exercises with emphasis on motor training for 12 months and daily home exercises.	BoNT treatment and regular PT 1x/week	TWSTRS (total, severity, disability, and pain scale), BDI, BAI, SF-36, CDQ-24	/	Both specialized PT and regular PT improved on the TWSTRS disability scale and general health over time.	No (Compared two type of PT)
Useros-Olmo et al., 2018	Case-control	27 (M = 5, F = 22)	Intervention 14,2 ± 9.5; control 11 ± 4.5	Four sessions of relaxation therapy of aquatic therapy (50 min) and autogenic training (15 min) at ratio of 1x/week with daily home exercises of autogenic training over 1 month.	No intervention	QoL (SF-36), TWSTRS (pain), VAS, BDI-II, state-trate anxiety (STAI)	/	Significant reduction in the PT group of pain, improved QoL with improved physical and mental health	No (not RCT)
Tassorelli et al., 2006	RCT	40 (M = 13, F:27)	6.9 ± 2.69	BoNT + PT daily 60 to 90 min sessions specific PT of stretching, exercises and biofeedback training for 2 weeks.	BoNT alone	Tsui scale, TWSTRS (total and pain scale)	/	Significant reduction in disability scale and subjective pain scores, lower BoNT dose is needed.	Yes
Stanković et al., 2017	RCT	14	13.5 ± 6.4 months	BoNT + PT The PT was given five times a week in the period of two weeks.	BoNT alone	TWSTRS (pain and disability), Tsui scale	6 months	PT combined with BoNT brought significant effect for pain, disability reduction.	No (Compared PT vs. PT with BoNT)
Smania et al., 2018	case series	4 (M = 1, F = 3)	21.2 months	30 PT sessions of one hour consisting of 20 min elongation techniques, 40 min of active postural reeducation at ratio of 4x/week during 6 weeks	1 h sessions of EMG biofeedback at ratio of 5x/week during 6 weeks	HRT, DQ, VAS	9 months.	Significant reduction of daily life disability and pain in both groups. Head-trunk alignment improved in both groups	No (not RCT)
Queiroz et al., 2012	Case-control	40 (M = 20, F = 20)	Median disease duration in intervention and control group is 9 (2.7–16) and 16 (6.5–17.5) respectively.	BoNT + PT sessions of 1h15 including motor learning exercise, stretching, active and passive mobilization of the cervical spine (1 h)/ and FES (15 min) for four weeks, five days a week, one hour and 15 min per session.	BoNT injection	TWSTRS (severity, disability, and pain) and QOL (SF-36)	/	Significant decrease of disease severity in both groups, PT group showed also a sig.decrease in pain, disability and improvement in physical and mental health	No (not RCT)
Pelosin et al., 2013	RCT	14 (M = 6, F = 8)	9.2 (± 6.3)	Kinesiotape on the dystonic SCM 14 days intervention with a new tape every 4days	Sham tape	VAS for pain, TWSTRS, somatosensory evoked potentials	/	45% reduction of pain after kinesiotape and no effect of tape on disease severity. 20% reduction of STDt in the affected body part	No (compared Kinesiotape vs. sham tape)
Hu et al., 2018	RCT	PT group were 8 (M = 4, F = 4), no PT group were 8 (F = 5, M = 3) and 10 controls	14.4 (± 10.9)	BoNT + PT including 1 session of deep massage, muscle elongation, stretching, instruction for home based exercises (stretching, ROM exercises) 15 min/day, 5x/week, for 6 weeks with weekly telephone calls	BoNT alone	TWSTRS (total, disability, and pain), VAS, SF-36	6 weeks	30% reduction of TWSTRS severity and pain in the PT group after intervention compared to the control group, changes in sensorimotor plasticity after BoNT with PT correlated with changes on TWSTRS whereas the control group showed no differences	No (reported the parameter of outcome differently)
Counsell et al., 2015	RCT	110 (M = 69, F = 85)	Intervention: 13,7 (± 9.3); control: 13,4 (± 7,4)	BoNT + Physiotherapy according to the Bleton technique 45 min sessions with home exercises, during 24 weeks at ratio of 1x/week	BoNT + standard PT care	TWSTRS, CDIP-58, Global impression of change, QoL (EQ-5D)	28 weeks	No difference between the improvement in the intervention or control group both interventions reduces disease severity and non-motor symptoms	No (compared two type of PT)
Castagna et al., 2019	Case-series	14 (M = 7, F = 7)	6 (± 7)	BoNT + PT 45 min sessions of passive mobilization (10 min) and Active sensorimotor exercises with augmented feedback (35 min), 18 at ratio of 3x/week	BoNT alone	TWSTRS, self-rating anxiety scale (SAS), BDI-II, and QoL (EQ-5D-5 L)	/	Total TWSTRS score was lower, no effect on anxiety, depression or quality of life	No (not RCT)
Boyce et al., 2013	RCT	20 (M = 6, F = 14)	10.2 (± 7.9)	8 PT sessions during 12 weeks, 30 min sessions of neck exercises, motor relearning and whole body relaxation with home exercises 4x/week	Whole body relaxation, with home exercise program	TWSTRS (total, disability, severity, and pain), CDQ-24, BDI-II, active cervical ROM measures	4 weeks	Significant reduction on TWSTRS, BDI and improvement of cervical ROM in both groups.	No (compared two type of PT)
Zetterberg et al., 2008	Case-series	6 (M = 4, F = 2)	/	PT for 4 week, 45 min sessions of stretching, endurance and strength exercises, muscle relaxation, postural orientation, 9x/week (2x/day except for 1 day)	None	CDQ-24, cervical dystonia postural orientation index (POI), Movement energy index (MEI), VAS, TWSTRS,	6 months	Significant improvement of pain, disease severity, disablity and QoL which remained after 6 months follow-up	No (not RCT)
Dec-Ćwiek et al., 2022	RCT	19 (M = 4, F = 15)	27.8 ± 12.4	BoNT + kinesiotape were performed for four consecutive weeks once per week.	BoNT +/- Sham taping	TWSTRS (Total, severity, disability, and pain scale) and CDQ-24	12 weeks therapy in each Experimental therapy.	Kinesiotape had no additional effect on the disease severity of CD but might improve QoL.	No (compared Kinesiotape and sham tape

### Risk of bias in studies

The overall risk bias was found to be ‘low’ for almost all RCTs [24, 26, 27, 29, 31, 32]; two RCTs [28, 30] had ‘some concerns’ about their quality. The methodological qualities of all case-control studies [25, 33, 34] were found to be good. The three case-series [35–37] have good quality (Tables 3, 4 and 5).

**Table 3:** Quality appraisal of randomized trials using Cochrane risk-of-bias tool for randomized controlled studies

References	D1	D2	D3	D4	D5	Overall
Van den Dool J. et al., 2019	Low	Low	Some	Low	Low	Low
Tassorelli et al., 2006	Low	Low	Low	Some	Low	Low
Stanković I. et al., 2017	Some	Some	Low	Low	Low	Some
Pelosin et al., 2013	Low	Low	Low	Low	Low	Low
Hu W. et al., 2018	Low	Some	Low	Low	Some	Some
Counsell C. et al., 2015	Low	Low	Some	Low	Low	Low
Boyce et al., 2013	Low	Low	Low	Low	Low	Low
Dec-Cwiek M et al., 2022	Low	Low	Low	Low	Low	Low

D1: Randomization process; D2: Deviations from the intended interventions; D3: Missing outcome data; D4: Measurement of the outcome; D5: Selection of the reported result

**Table 4:** Quality appraisal of non-randomized experimental studies using JBI risk-of-bias tool

Criteria	Useros-Olmo et al., 2018	Queiroz, M. A. et al., 2012	Werner C et al., 2019
Is it clear in the study what is the ‘cause’ and what is the ‘effect’	Y	Y	Y
Were the participants included in any comparisons similar?	Y	Y	Y
Were the participants included in any comparisons receiving similar treatment/care, other than the exposure or intervention of interest?	Y	Y	Y
Was there a control group?	Y	Y	Y
Were there multiple measurements of the outcome both pre and post the intervention/exposure?	N	Y	Y
Was follow up complete and if not, were differences between groups in terms of their follow up adequately described and analyzed?	Y	N	N
Were the outcomes of participants included in any comparisons measured in the same way?	Y	Y	Y
Was appropriate statistical analysis used?	Y	Y	Y
Were outcomes measured in a reliable way?	Yes	Y	Y
Overall appraisal (Include, exclude, seek further info)	Include	Include	Include

Options for rating: Yes/no/unclear/not applicable

**Table 5:** Quality appraisal of case-series studies using JBI risk-of-bias tool

Criteria	Zetterberg L. et al., 2008	Smania, N. et al., 2018	Castagna et al., 2019
Were there clear criteria for inclusion in the case series?	Y	Y	Y
Was the condition measured in a standard, reliable way for all participants included in the case series?	Y	Y	Y
Were valid methods used for identification of the condition for all participants included in the case series?	Y	Y	Y
Did the case series have consecutive inclusion of participants?	Y	Y	Y
Did the case series have complete inclusion of participants?	Y	Y	Y
Was there clear reporting of the demographics of the participants in the study?	Y	Y	Y
Was there clear reporting of clinical information of the participants?	Y	Y	Y
Were the outcomes or follow up results of cases clearly reported?	Y	Y	Y
Was there clear reporting of the presenting site(s)/clinic(s) demographic information?	Y	Y	Y
Was statistical analysis appropriate?	Y	Y	Y
Overall appraisal (Include, exclude, seek further info)	Include	Include	Include

Options for rating: Yes/no/unclear/not applicable

### Result of individual studies

#### Effect of physical therapy on pain

The effect of PT on pain was investigated in all of the 14 studies [24–37]; four studies reported the VAS scale [28, 29, 35, 36]; 10 studies [24–28, 30–32, 34, 37] reported pain by the pain subscale of the TWSTRS; one study [33] reported both. All included studies reported a reduction of pain after physical therapy intervention which was 30% [28, 36], 40% [34], and up to 50% [24] larger in the PT group compared to the control group. When 2 types of PT intervention are compared, both types resulted in reduction of pain, no differences between the interventions were found for pain [27, 31].

#### Effect of physical on Disease severity

The effect of PT on disease severity was investigated in 7 studies by the TWSTRS [25, 26, 28, 30–32, 34]. Van den Dool et al. found an improvement of dystonic postures in the intervention group after they received specialized PT which emphasized motor training [31]. Similarly, other studies reported statistically significant improvement in severity scores in the PT group [28, 32].

#### Effect of physical therapy on disability

The effect of PT on disability was investigated in seven studies [25, 26, 28, 30–32, 34] by the TWSTRS and reported significant improvements. Tassorelli et al. also reported a marked reduction in disability score by 4.5 in activities of daily living as compared to BoNT therapy alone [24]. When 2 types of PT intervention are compared, both types resulted in reduction of disability of 1.7 points on the TWSTRS subscale [31]. However, no differences between the interventions were found [27, 31].

#### Effect of physical therapy on QoL

The effect of PT on patient QoL was investigated in 10 studies [25–28, 31–34, 36, 37]. It was measured by the Short Form Health Survey (SF-36) in four studies [25, 28, 33, 34], three studies [26, 32, 36] used the Craniocervical Dystonia Questionnaire (CDQ-24), two studies [27, 37] used the EuroQol group quality questionnaire (EQ-5D), and one study used both the SF-36 and CDQ-24 [31]. Data on QoL were not always clearly reported. There is conflicting evidence on the different domains of the SF-36. General health perceptions improved after PT intervention in two studies [31, 34], mental health improved in three studies [25, 33, 34] but one study found no effect [28].

### Meta – analysis: effect of PT additional to BoNT

**Fig. 2:**
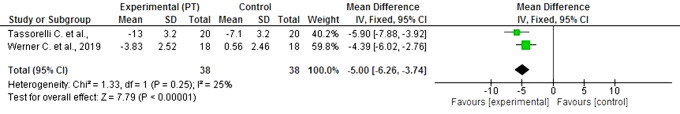
Forest plot of meta-analysis of the effect of physiotherapy on TWSTRS pain score

Two studies with low risk of bias reporting on the effect of PT additional to BoNT compared to BoNT alone were selected [24, 25]. A fixed effect model was used, and the mean difference was reported. Regarding study heterogeneity, there was no evidence of clinical, methodological, or statistical heterogeneity for outcome analysis of pain. For the other outcome measures of disease severity, disability, and QoL, there was significant heterogeneity observed among the studies. The I^2^ statistics were greater than 50% and the *p*-value of test of the overall effect of Z was found to be > 0.05 in all those parameters. So a meta-analysis could only be done for the outcome parameter of pain. Pooling the data of the two studies revealed that PT intervention for CD patients as an add-on therapy had a significant positive effect on pain measured by the TWSTR pain scale (-5.00, 95% CI -6.26, -3.74) (Fig. 2).

## Discussion

Given the progress in the field since the previous literature review in 2014, new emerging evidence on the effectiveness of PT for cervical dystonia has been published. An update of the knowledge was therefore warranted. Of the 14 articles included, 8 were RCTs. The literature shows that PT has a beneficial effect on different outcomes such as pain, disability, and disease severity. The effect on QoL is conflicting.

The finding of the meta-analysis revealed a positive effect of PT for reducing pain. BoNT in itself is beneficial on different outcomes such as pain and disease severity [11]. However, when PT is added to BoNT interventions, the pooled findings of two studies [24, 25] showed a reduction in pain (-5.00, 95% CI -6.26, -3.74) in groups receiving multimodal PT intervention with BoNT compared to BoNT alone. Pain reduction was reported in all included studies so we recommend physical therapy additional to BoNT for pain reduction in patients with CD. A 6 week PT intervention results in decreased pain and disease severity which correlated to enhanced sensorimotor plasticity. This was reported in the study of Hu et al. [28] in CD and previously in patients with writers’ cramp [38]. The central changes following physical therapy may account for the improvement in motor function and reduction of pain [39]. But the working mechanism need to be further explored.

Regarding the effect of physical therapy on disease severity and disability and quality of life, improvements are reported in 7 studies [24, 25, 30, 31, 34–36]. However, the heterogeneity between the studies was too high to pool the data so no firm recommendations can be made. Nevertheless, clinicians and patients might expect improvements in disease severity and disability following a physical therapy program.

A broad variety of PT intervention modalities were used in the included studies. Two studies used active exercises exclusively with relaxation therapy and motor relearning exercises [26, 33], 2 studies solely applied kinesiotape [29, 32], and 10 used combined modalities [24, 25, 27, 28, 30, 31, 34–37]. The PT program used by Tassorelli et al., included passive myofascial elongation manoeuvres, deep massage of cervical muscle, biofeedback training, and active stretching of muscles, tendons, and ligaments [24]. The main PT techniques by Werner et al. consisted of passive and active mobilization of the cervical spine and shoulder girdle, feedback exercises, perception and coordination training, posture training, and relaxation [25]. In one RCT of Hu et al., subjects were trained to perform a 6 week home exercise program after one supervised PT session [28]. These home exercises resulted in a 30% reduction of the TWSTRS score, indicating the benefits of exercising at home, or the possible use of telemedicine in CD. In the studies by Counsell et al. and Van den Dool et al., standard PT interventions were compared to a specialized PT program [27, 31] with no clear favorable outcome for the specialized interventions. Despite the large variability in intervention modalities in the intervention group, patients receiving PT were positively favoured over the control group. In line with this, existing evidence highlighted the modulating effect of exercise on abnormal movement patterns in healthy adults and neurological disorders [39, 40]. However, there is a lack of insight in dystonia.

Two RCTs reported pain reduction after applying KinesioTape [29, 32]. This effect was studied on short term (2–4 weeks) and may be attributed to changes in somatosensory temporal discrimination [29]. Applying tape did not have an impact on disease severity so it seems that active exercises are necessary to obtain these changes.

The findings of this literature review are in line with the previous review of 2014 but with higher quality studies included, enabling a meta-analysis. The findings are also in line with the review of Loudovici-Krug [41], which included 6 RCTs of 2 databases. The current review is a more comprehensive review including 8 RCTs with a more comprehensive overview of the effect of PT on several disease outcomes such as pain, disability, severity, and quality of life.

Based on the findings in this review, we cannot state the difference in effect size of PT alone compared to BoNT alone. BoNT alone has beneficial effects on pain and disease severity [11]. When combined with PT, the reduction in pain, disease severity and disability is even larger, based on our results. But to our knowledge, no research is available comparing the effect of BoNT alone to PT alone as BoNT is the first treatment of choice.

### Implications for future research

The diverse nature of the comparison groups in the care they were receiving did not allow the reviewers to quantify the impact of PT on the outcomes such as TWSTRS total, disability, severity, and QoL. Thus, there is a need for further high-quality RCTs with a large study population that considers the comparison of PT with BoNT and BoNT alone. Up to now, it is unclear how long PT interventions should last before reaching optimal effect on neuroplasticity and lead to optimal treatment outcome for patients. Further fundamental research might address the underlying mechanisms in the sensorimotor network.

### Implications for clinical practice

Various PT intervention modalities with different duration and frequencies were used in included studies. All studies showed a decrease in pain intensity. A clear recommendation of a specific type of PT as a favourable therapeutic intervention in the clinical setup cannot be made but multimodal interventions show effect on multiple outcome parameters.

### Strengths and limitations of the review

As strength, the methodological quality of included articles was assessed independently by two reviewers. Furthermore, a meta-analysis was done with strict inclusion criteria.

As a review limitation, we searched articles published in the English language from three databases only. Three articles reported the outcome parameter of interest in a different way and were excluded in some of the outcome analysis. Furthermore, the limited number of primary studies with heterogeneous findings did not allow to perform meta-analysis for all outcome measures.

## Conclusions

We recommend physical therapy interventions additional to BoNT for improving pain in patients with cervical dystonia. Patients might expect improvements in disease severity, disability and QoL.

Overall, the current review findings indicate beneficial effects of PT in reducing disease severity, disability, pain, and improving patient QoL. The meta-analysis also showed the statistically significant positive effect of PT in reducing TWSTRS pain scores. However, there is a lack of evidence to recommend the preferred type of PT, its duration, and frequency.

Given the variety of PT modalities used, no clear recommendation can be formulated on the best possible practice. Thus, there is a need to conduct additional RCTs before making a clear recommendation on the content of the therapy.

### Protocol registration

This review protocol was registered in PROSPERO with the number CRD42022376433.

## Data Availability

All data generated and analyzed during this review are included in this manuscript.
